# A portable system for collecting anatomical joint angles during stair ascent: a comparison with an optical tracking device

**DOI:** 10.1186/1476-5918-8-3

**Published:** 2009-04-23

**Authors:** Jeroen HM Bergmann, Ruth E Mayagoitia, Ian CH Smith

**Affiliations:** 1 Division of Applied Biomedical Research, King's College London, London, UK

## Abstract

**Background:**

Assessments of stair climbing in real-life situations using an optical tracking system are lacking, as it is difficult to adapt the system for use in and around full flights of stairs. Alternatively, a portable system that consists of inertial measurement units (IMUs) can be used to collect anatomical joint angles during stair ascent. The purpose of this study was to compare the anatomical joint angles obtained by IMUs to those calculated from position data of an optical tracking device.

**Methods:**

Anatomical joint angles of the thigh, knee and ankle, obtained using IMUs and an optical tracking device, were compared for fourteen healthy subjects. Joint kinematics obtained with the two measurement devices were evaluated by calculating the root mean square error (RMSE) and by calculating a two-tailed Pearson product-moment correlation coefficient (r) between the two signals.

**Results:**

Strong mean correlations (range 0.93 to 0.99) were found for the angles between the two measurement devices, as well as an average root mean square error (RMSE) of 4 degrees over all the joint angles, showing that the IMUs are a satisfactory system for measuring anatomical joint angles.

**Conclusion:**

These highly portable body-worn inertial sensors can be used by clinicians and researchers alike, to accurately collect data during stair climbing in complex real-life situations.

## Background

In terms of self-rated health, the most important activities of daily living are those involving mobility [1]. Self-reported difficulty in stair climbing has shown to be useful in assessing and defining functional status of older adults [2]. Obtaining accurate data about mobility is therefore of great clinical relevance and could lead to further improvements in various rehabilitation treatments [3]. Compared to level walking only a limited number of studies have investigated the kinematics and kinetics of normal stair climbing [4-11]. In general, kinematics and biomechanical aspects of stair climbing are studied using laboratory staircases combined with an optical motion analysis system [4,11]. Although this kind of research yields valuable information, the results only remain valid in conditions where no anticipation or reaction to a real-world environment is required. In addition, it is almost impossible to use any form of optical tracking on stairwells, as the vertical shaft which contains the staircase limits the placement of cameras. Collecting data during stair climbing in a more real-life, complex environment requires a portable and lightweight measuring device. Zhou et al (2006) showed that inertial measurement units (IMUs) consisting of gyroscopes, accelerometers and magnetometers used to measure upper limb motion can accurately estimate arm position [12]. Accelerometers and gyroscopes have also been proven to be able to correctly record shank, thigh and knee angles during level walking and a variety of lower leg exercises [13,14]. Although, a miniature gyroscope attached to the shank is able to detect different cycles during stair ascent [15] and position data of the foot can be gathered with the combination of a gyroscope and two accelerometers [16], a portable system that can collect anatomical joint angles during stair climbing has not yet been reported.

The purpose of this study is to compare the anatomical joint angles determined by IMUs during stair ascent, to those joint angles acquired with an optical tracking device. Measuring stair climbing can be of great clinical relevance, as according to the Canadian Institute for Health Information the most common specified type of falls (23%) for people of 65 years and over are falls on or from stairs and steps [17]. Furthermore, it has also been shown that, for certain patient groups, stair climbing can be a more critical pre-clinical assessment than walking [18].

## Methods

Fourteen healthy subjects, nine men and five women, with a mean age of 27 years (range 20 to 37) voluntarily participated in this study. Their mean (± standard deviation) height and weight were 175 (± 8) cm and 69 (± 10) kg. The protocol was approved by the College Research Ethics Committee. All subjects gave written informed consent before the experiment. Each subject was asked to ascend a staircase consisting of four steps during twelve separate trials. Subjects were instructed to climb the stairs in the way they felt most comfortable. Each step was 62 cm wide, 23 cm long and 15 cm high giving the stair a pitch angle of 31 degrees. The subject stood in front of the stair and started ascending the stair when a verbal signal was given.

**Figure 1:**
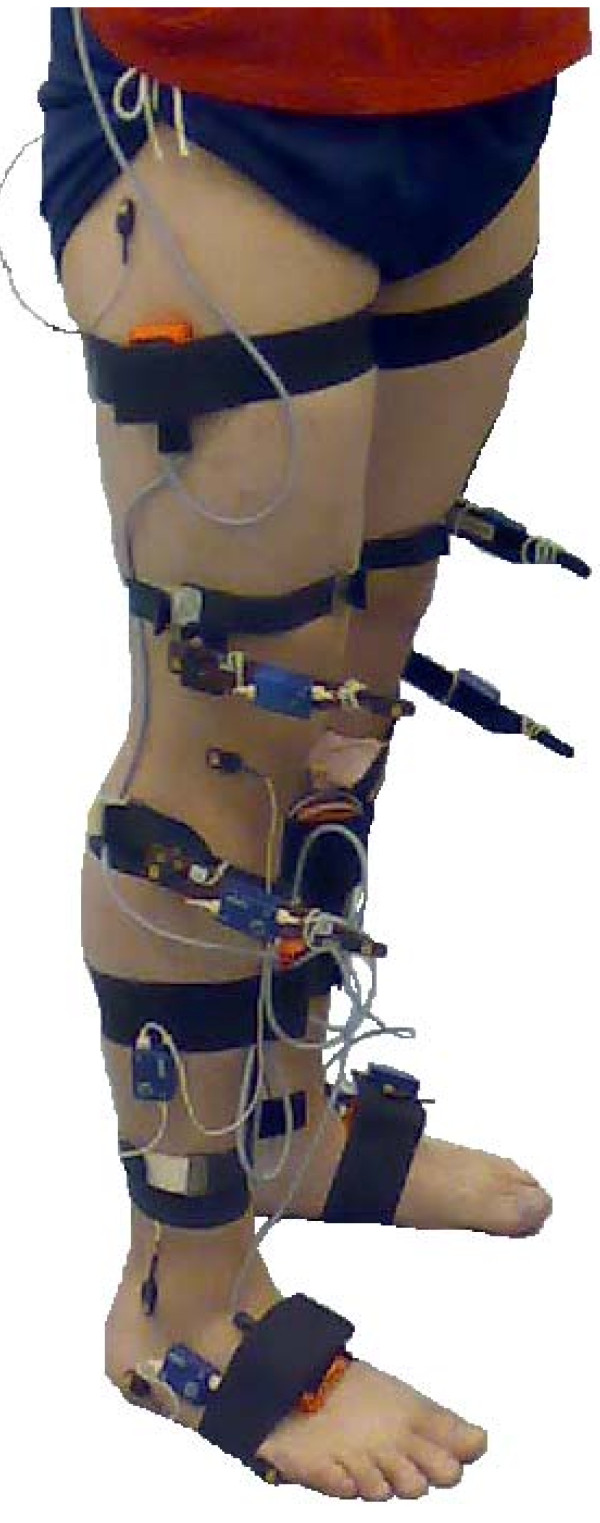
**Sensor set up used during study**. Optical tracking markers and Inertia Measurement Units (IMUs) as attached to each subject.

Six IMUs (MTx, Xsens Technologies B. V., Enschede, Netherlands) were placed on the dorsal side of both forefeet [19], halfway up the medial surface of the tibias [19] and two thirds up the tensor fascia latae of each leg using double-sided adhesive tape with additional elastic straps to hold them in place (Figure 1). Straps were used to provide a preloading force and thereby decreasing measuring errors [20]. The sensors were securely attached to each body segment in order to assure that the orientation of the sensor with respect to the body segment did not change. Observations made during a pilot study indicated that the current positions used for sensor placement, minimized relative motion between sensor and underlying bones.

During static stance, the X-axis of each IMU coordinate system was physically placed to be in the sagittal plane after an analytically alignment of the axes by software (MT Software V2.8.1, Xsens Technologies B. V., Enschede, Netherlands). The software program placed the Z-axis of each IMU in line with gravity (vertical plane) with the new X-axis of the sensor perpendicular to the Z-axis and along the line of the original X-axis [21]. The non-orthogonality between the axes of the body-fixed co-ordinate system is less then 0.1° [21].

Active Codamotion (Codamotion, Charnwood Dynamics, Leicestershire, UK) markers were placed (Figure 1) on the toe (5th metatarsal head), ankle (lateral malleolus), knee (fibula head and lateral femoral condyle), hip (trochanter major) and on the side of stairs. These markers were fixed using double-sided adhesive tape. The Bilateral Segmental Gait Analysis system configuration was used for data acquisition by the Codamotion and Motion Tracker software. The cameras of the optical tracking device were positioned in such a way, that the position data of the markers on the right side could always be obtained during stair ascent. Data for both the Codamotion and the IMUs was acquired at 100 Hz and an electronic pulse was used to synchronize the two measurement devices. All further data analysis was done using Matlab (MathWorks, Inc, Natick, Massachussetts, USA).

### Data analysis

The lower extremity could be approximated as a multi-link chain, with each body part as a rigid segment represented by one IMU [22]. Only movements around the transverse axis (resulting in flexion-extension kinematics) were studied, as the largest range of motions of the lower extremity occur around this axis during stair climbing [7].

The rotation matrix (R_DCM_), which was acquired from each IMU, was used to determine the Euler angle (*θ*) that represented rotation around the transverse axis. This angle is calculated by combining the value A_31 _obtained from the IMU with the element in row three, column one of the Euler sequence ().

(1)

(2)

If equation 1 and 2 are combined, then;

(3)

**Figure 2:**
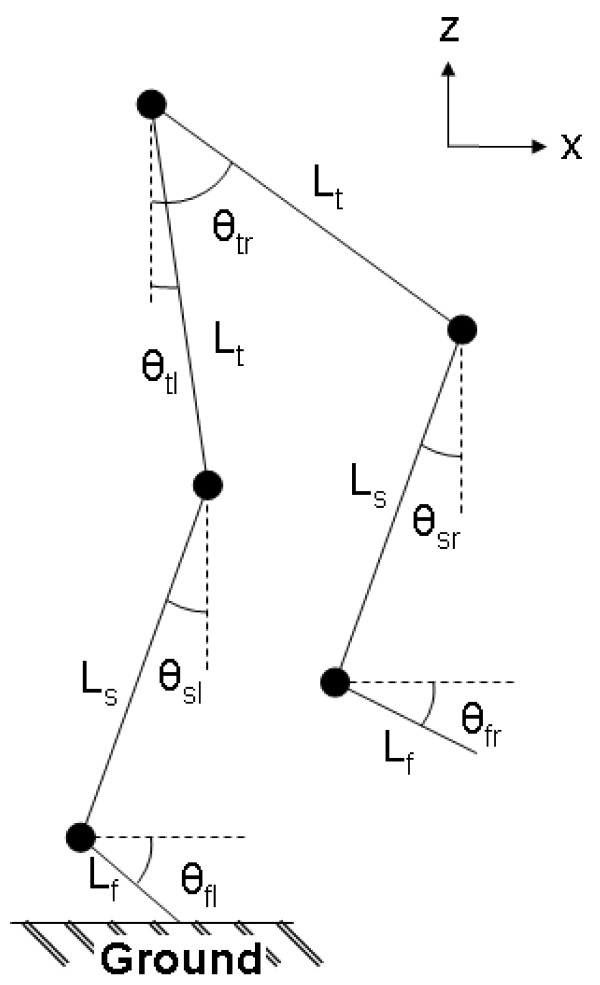
**Six-link sagittal model**. Segment lengths were taken from anthropometric data [22]. (L_f_) length of the foot; (L_s_) length of the shank; (L_t_) length of the thigh; (*θ*_fl_) angle of the left foot; (*θ*_sl_) angle of the left shank; (*θ*_tl_) angle of the left thigh; (*θ*_fl_) angle of the right foot; (*θ*_sl_) angle of the right shank; (*θ*_tl_) angle of the right thigh.

The angle (*θ*) for each of the six IMUs combined with segment lengths of the foot, shank and thigh were used in a six-link sagittal model (Figure 2). The segment lengths were calculated from anthropometric data [22], which was a percentage of the body height of each subject in order to keep the model simple.

The knee angle (*α*) was determined with the IMUs, by subtracting the angle around the transverse axis of the shank from that of the thigh. The flexion-extension angle of the ankle was found by subtracting the lower leg angle from the foot angle in the sagittal plane, while the thigh angle was represented by the upper leg angle with respect to the vertical axis [23].

For the optical tracking device the knee angle was defined as the angle between a spatial vector joining the lateral malleolus to the fibula head and a spatial vector joining the lateral femoral epicondyle to the greater trochanter [24]. The equations used are directly taken from [24] and the computations were carried out in three steps,

(4)

(5)

(6)

In which *x*_6_, *y*_6_, *z*_6 _are spatial coordinates of the trochanter major; *x*_5_, *y*_5_, *z*_5 _are spatial coordinates of the epicondylus fermoris lateralis; *x*_4_, *y*_4_, *z*_4 _are spatial coordinates of the head of the fibula; *x*_3_, *y*_3_, *z*_3 _are spatial coordinates of the malleolus lateralis. L_65 _is the length between the epicondylus fermoris lateralis and the trochanter major, while L_43 _is the distance between the head of the fibula and the malleolus lateralis. This calculation method described by Kiss, Kocsis and Knoll determines a knee angle (*α*) which only depends on the relative position of the shank to the thigh [24].

The same calculation can be applied for the determination of the ankle angle (*β*), by using the spatial coordinates of the most lateral aspect of the calcaneus (*x*_2_, *y*_2_, *z*_2_) and the 5th metatarsal head (*x*_1_, *y*_1_, *z*_1_) instead of those of the epicondylus fermoris lateralis and the trochanter major and by replacing the distance between the head of the fibula and the malleolus lateralis with the distance between the most lateral aspect of the calcaneus and the 5th metatarsal head. The thigh angle (*γ*) was defined as the angle between a spatial vector joining the epicondylus fermoris lateralis and the trochanter major and a vertical spatial vector.

Time was converted to percentages, starting from the onset of movement until the top of the stairs was reached, to allow accurate comparisons within subjects. All angles were normalized in time per trial and subject by calculating the mean angle per percentage of time.

### Statistical analysis

Data was normally distributed as observed in the probability plots and histograms. All anatomical joint angles on the right leg, obtained with the two measurement devices, were evaluated by calculating a two-tailed Pearson product-moment correlation coefficient (r) and by calculating the root mean square error (RMSE) between the two signals [14,25]. A paired t-test was used to compare the maximum range of motion obtained by IMUs with those obtained by the optical tracking device per subject (n = 14) and to determine if the slopes of the linear regressions differed from one. The significance level was set at 0.05.

## Results

**Figure 3:**
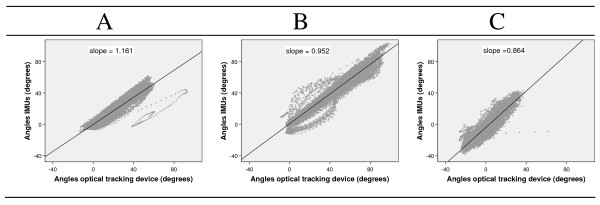
**All angles obtained by the optical tracking device plotted against those of the IMUs**. Slopes of the linear regression between the two variables are displayed for each graph. A: Ankle, B: Knee, C: Thigh.

The relationships between anatomical angles obtained by the two measurement devices were shown to be linear (Figure 3). The slopes for the ankle and thigh each differed by about 15% from unity (p < 0.01), whilst the slope for the knee did not (p = 0.10).

**Figure 4:**
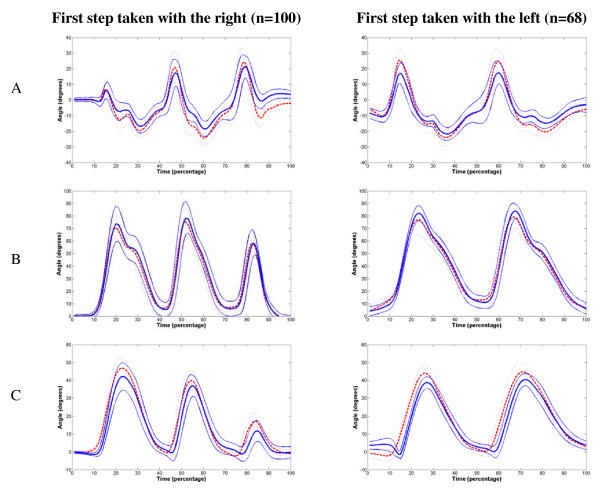
**Mean angles and standard deviation of the right leg in the sagittal plane**. Thick red dotted lines are the mean angles obtained by IMUs and the thick blue solid lines are those obtained by the optical tracking device. Thin lines represent the standard deviations. A: Ankle, B: Knee, C: Thigh.

During a hundred trials subjects took their first step with the right foot, while they started with the left in 68 trials (Figure 4). Seven subjects started all trials with the right foot, three constantly started with the left and four participants alternated between left and right foot. It was observed that the IMUs on average had higher peak values at the ankle and thigh compared to the optoelectronic system, while the opposite was found for the knee (Figure 4).

A significant difference was found between the maximum range of motion of the ankle and thigh (p < 0.01) obtained with the IMUs compared to those acquired with the optical tracking device (Table 1). No difference was found for the maximum range of motion at the knee joint (p = 0.47).

**Table 1:** Pearson correlations, Root Mean Square Errors and maximum Range of Motions

	*Pearson correlation coefficient* *(p < 0.01) *Mean (SD)	*Root Mean Square Error in degrees *Mean (SD)	*Maximum Range of Motion in degrees* Mean (SD)	
			
			IMUs	Optical device
Ankle angles	0.93(± 0.05)	4 (± 2)	63 (± 8) *	54 (± 8)

Knee angles	0.98(± 0.05)	4 (± 3)	91 (± 8)	92 (± 6)

Thigh angles	0.96(± 0.06)	5 (± 3)	56 (± 5) *	49 (± 4)

Mean correlations and Root Mean Square Errors between joint angles in the sagittal plane, acquired by the IMUs and the optical tracking device over all the 14 subjects and 12 trials (n = 168). Maximum Range of Motion obtained with both measurement devices per subject (n = 14). SD: standard deviation. Asterisks indicate difference (p < 0.01) between maximum Range of Motion with respect to the optical tracking device.

## Discussion

The aim of the study was to investigate if the anatomical joint angles determined by IMUs sufficiently approximate the anatomical joint angles that were gathered with an optical tracking device. Strong correlations and mean RMSE of 4 to 5 degrees were found for all angles, comparable to those obtained using a similar system to track upper limb motion [26]. Similar correlations were also found for linear acceleration trajectories obtained from IMUs, when compared with those derived from optical tracking position data [25]. Yet, in this study the mean RMSE as a percentage of the maximum value (4 to 9 percent) was higher than the percentages found for the linear acceleration trajectories, which were in the region of 1 to 6 percent.

In the unpublished pilot study to this paper, which compared the equipment component of both systems, Pearson's correlation coefficients of 0.999 (p < 0.001) between the IMUs and optical tracking device were found, with a RMSE of 1°. As the RMSE during stair climbing (4–5°) was greater than the RMSE found in the pilot study (1°), a small misalignment between the two coordinate systems could have been present. This misalignment might be explained by the fact that the IMUs were placed in the middle of the body segments, whilst the active markers of the optical tracking device were positioned on bony landmarks. However, these locations were chosen in order to minimize any motion artefact. Despite optimizing placement of markers, both systems suffer from motion artefacts. A translational displacement between bony landmark and marker is likely to occur during stair climbing, causing errors in estimating position during movement [27,28], which in turn leads to inaccuracies in determining angles. The IMUs measure orientation rather than position and are consequently less prone to errors caused by translational displacement of the sensors. Errors related to movement can however still occur in the IMUs, because of rotational displacement of the sensor relative to the body segment, due to for example change in muscle contour. Future research is needed to investigate to what extent the IMUs are prone to this kind of error.

High accelerations can easily lead to an increase in errors, as many IMUs use the accelerometer unit as an inclinometer [29]. If the magnitude of the acceleration can no longer be neglected with respect to the gravity, the accuracy of the orientation measurement will be reduced, making this kind of IMUs unsuitable to measure human movements during which high accelerations are occurring.

The range of motion, measured with the optical tracking device, at the ankle (54° ± 8) and the knee (92° ± 6) was comparable to the range (respectively, 56° ± 7 and 86° ± 5) observed by Mian et al (2007) in young adults climbing stairs [7]. However, the range of motion at the thigh (49° ± 4) was higher then the range (30° ± 4) reported by Mian et al (2007), which might be related to a difference in the method used to calculate joint angles, at the hip, from optical tracking data.

Maximum range of motion of the knee angle was similar between the two measurement devices, but did differ in the thigh and ankle angles. Any inaccuracies in range of motion of the ankle angles were further increased by taking the foot as a single rigid segment, as motion occurs between the different parts of the foot [30]. The fact that the foot is multi segmental, might explain why an IMU placed on the dorsal part of the foot provides a different range of motion compared to the optical markers placed on the heel and the toe. Some further clues about the differences found between the two systems are provided by inspection of individual traces which deviate strongly from the rest (Figure 3). Data inspection showed that these deviations occurred in the optical tracking marker position, presumably due to movement of the marker with respect to the bony landmark.

Activities of daily living have been previously investigated using mobile sensors, consisting of uniaxial accelerometers [3]. More recently, inertial sensors, similar to those used in this study, were utilized to track upper limb motion without showing any notable drift in the estimation of the movements [26]. No significant drift problems arose during data collection in this study, although future work is needed to determine how well the proposed method works during longer data collection periods.

## Conclusion

In general, IMUs provide a good alternative for measuring joint angles of the lower extremity during stair ascent when compared to positional markers. In addition, they provide the opportunity to perform accurate measurements in complex real-life environments using a non constraining measurement device. Furthermore, the IMUs were easy to set up, giving rise to the opportunity for clinicians and researchers to measure stair climbing out of the laboratory setting.

## Competing interests

The authors declare that they have no competing interests.

## Authors' contributions

JB conceived the study, participated in study design, data collection, calculation, and statistical analysis, drafted and revised manuscript. RM conceived the study, participated in study design and revised manuscript. IS conceived the study, participated in study design, and statistical analysis, revised manuscript and gave final approval of the version to be published. All authors read and approved the final manuscript.
